# Serum microRNA analysis facilitates decision-making between active surveillance and immediate surgery for low-risk thyroid tumors

**DOI:** 10.20945/2359-4292-2025-0072

**Published:** 2025-08-18

**Authors:** Fernanda Nascimento Faro, Jacqueline Montalvão Araújo, Mariana Mazeu Barbosa de Oliveira, Antônio Augusto Tupinambá Bertelli, Pedro Ivo Ravizzini, Laura Sterian Ward, Nilza Maria Scalissi, Adriano Namo Cury, Rosália do Prado Padovani, Carolina Ferraz

**Affiliations:** 1 Centro de Tireoide, Serviço de Endocrinologia, Departamento de Medicina, Irmandade da Santa Casa de Misericórdia de São Paulo, São Paulo, SP, Brasil; 2 Serviço de Cirurgia de Cabeça e Pescoço, Departamento de Medicina, Irmandade da Santa Casa de Misericórdia de São Paulo, São Paulo, SP, Brasil; 3 Serviço de Radiologia, Departamento de Medicina, Irmandade da Santa Casa de Misericórdia de São Paulo, São Paulo, SP, Brasil; 4 Laboratório de Genética Molecular do Câncer, Departamento de Medicina, Universidade Estadual de Campinas, Campinas, SP, Brasil; 5 Serviço de Medicina Nuclear, Irmandade da Santa Casa de Misericórdia de São Paulo, São Paulo, SP, Brasil

**Keywords:** Thyroid neoplasms, Carcinoma, papillary, Watchful waiting, Circulating microRNA, Molecular diagnostic techniques

## Abstract

**Objective:**

To develop a practical and cost-effective test to distinguish patients with malignant thyroid nodules eligible for active surveillance from those requiring immediate surgery.

**Methods:**

This prospective observational study included patients with malignant thyroid nodules (3 to 15 mm) who were assigned to either an Active Surveillance Group (n = 30) or a Surgery Group (n = 21) based on the institutional protocol. The Surgery Group was further stratified according to the American Thyroid Association risk of recurrence/persistence. Preoperative serum levels of miR-146b-5p and miR-204, normalized to miR-16, were analyzed. Receiver operating characteristic curves were used to establish cut-off values to differentiate between low and intermediate/high risk of recurrence/persistence, which were subsequently applied to the Active Surveillance Group.

**Results:**

Patients were initially assigned to the active surveillance (n = 30; 53.5 ± 12.6 years old) or Surgery Group (n = 21; 41.9 ± 7.9 years old). The mean follow-up duration for the Active Surveillance Group was 36.4 ± 25.8 months, during which no patients experienced disease progression. Five patients in the Active Surveillance Group were subsequently transitioned to the Surgery Group. Molecular analysis of the Surgery Group indicated that upregulation of miR-146b-5p/miR-16 and downregulation of miR-204/miR-16 were significantly associated with intermediate/high risk of recurrence/persistence (p = 0.005 and 0.006, respectively). Downregulation of miR-204/miR-16 demonstrated a sensitivity of 75% and a negative predictive value of 86.7%. The combination of upregulation of miR-146b-5p/miR-16 and downregulation of miR-204/miR-16 yielded both a specificity and negative predictive value of 100%.

**Conclusion:**

Decision-making for patients with low-risk papillary thyroid carcinoma regarding eligibility for active surveillance can be facilitated through serum analysis of miR-204/miR-16 expression, which may be used as a rule-out test. In contrast, combined analysis of miR-146b-5p/miR-16 and miR-204/miR-16 can serve as a rule-in test.

## INTRODUCTION

The incidence of thyroid cancer has increased significantly over the past 30 years (^1,2^), primarily due to a greater number of diagnoses of small papillary thyroid carcinomas (PTC) (^3,4^). Given the evidence of PTC indolence and the risks associated with surgery (^5-11^), treatment de-escalation has been proposed, with active surveillance (AS) emerging as an alternative for low-risk disease, reserving surgery only for cases of disease progression. The American Thyroid Association (ATA) and the European Thyroid Association have recommended AS for thyroid nodules smaller than 1 cm (^12,13^); nonetheless, some authors have expanded AS protocols to include nodules up to 1.5 cm (^14-16^).

The lack of markers to identify tumor aggressiveness at the time of diagnosis remains a barrier to AS implementation. The BRAFV600E mutation, which is the most common mutation in PTC, does not appear to be associated with tumor growth or metastasis in small PTC, as it occurs at a high incidence even in patients without disease progression (^17,18^). Somatic mutations in the promoter regions of telomerase reverse transcriptase (TERT) predict a worse prognosis in PTC; however, their role in small tumors is uncertain, as they are rarely detected (^17-21^).

The expression of microRNAs (miRs) is currently the most promising biological marker of PTC, although little is known about their value in small PTCs (^22^). miRs are small molecules that negatively regulate gene expression at the post-transcriptional level of messenger RNA (^23^). Unlike mutations involved in the mitogen-activated protein kinase cascade (e.g., the BRAFV600E mutation), miRs act through multiple pathways and contribute to tumor pathogenesis by regulating oncogenes and tumor suppressor genes (^24,25^). Consequently, some miRs promote oncogene activity when overexpressed, whereas others exert a protective role, with their downregulation leading to disruption of tumor suppressor genes (^24,25^).

A wide range of miRs are associated with PTC. Overexpression of miR-146b-5p is highly accurate in distinguishing benign from malignant thyroid nodules and is frequently associated with invasion, lymph node metastasis, and tumor recurrence, serving as a marker of aggressive PTC behavior (^22,24,26,27^). In contrast, downregulation of miR-204 is associated with clinical and histological features of aggressive PTC (^28,29^).

miR expression can be evaluated in tissues, fine-needle aspiration cytology material, or blood. Analysis of miRs in blood, known as liquid biopsy, appears to be the most suitable method for AS screening. Its primary advantages include the high stability of miRNAs in blood and their equivalence to tissue expression, in addition to the practicality of sample collection (^30-33^).

Therefore, this study aimed to develop a test capable of predicting the behavior of low-risk PTC at diagnosis based on serum miR expression in order to facilitate decision-making regarding the indication for AS.

## METHODS

### Study design

**Figure 1:**
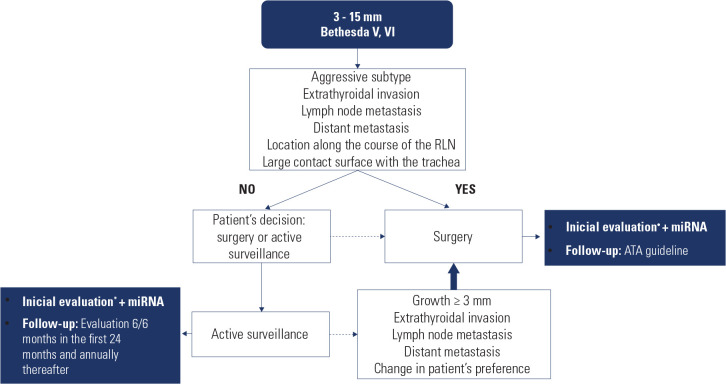
Study design.

Source: Haugen and cols. (^12^).

Figure 1 shows the design of this prospective observational study. All patients underwent an initial evaluation that included cervical and thyroid ultrasound, measurements of serum thyroglobulin, antithyroglobulin antibodies, thyroid stimulating hormone (TSH), free T4, and calcitonin levels, as well as the collection of blood samples for miRNA analysis (prior to any surgical intervention). The thyroid nodule was evaluated for the following characteristics: suspicion of extrathyroidal invasion, lymph node metastasis, extensive contact with the trachea, location along the course of the recurrent laryngeal nerve, clinical signs of distant metastasis, or cytology suggestive of an aggressive histological subtype. After this assessment, patients were informed about the possibility of AS as an alternative to immediate surgery.

* Initial evaluation: thyroid ultrasound, measurement of serum thyroglobulin, anti-thyroglobulin antibody, thyroid stimulating hormone, free T4, and calcitonin.

RLN: recurrent laryngeal nerve; ATA: American Thyroid Association.

Patients who opted for immediate surgery underwent either lobectomy or total thyroidectomy, as determined by the attending physician, and were followed up according to ATA guidelines (^12^). Patients who chose AS underwent follow-up examinations, including ultrasound and measurement of serum thyroglobulin, antithyroglobulin antibody, TSH, and free T4 every 6 months for the first 24 months and annually thereafter. Patients were referred for surgery if the nodule increased by over 3 mm in its largest diameter, demonstrated signs of extrathyroidal invasion, showed lymph node or distant metastasis, or if the patient changed their treatment preference.

The Surgery Group was classified according to the ATA risk of recurrence and/or persistence (RRP) as either lowor intermediate/high-risk (^12^). A Receiver Operating Characteristic (ROC) curve was used to establish cut-off values for miR expression capable of distinguishing between low and intermediate/high RRP. Based on these thresholds, rule-in and rule-out tests were developed, which were subsequently applied to the Active Surveillance Group (AS Group) using previously established cut-off values.

### Eligibility criteria

Patients were recruited using convenience sampling. Eligible participants were consenting individuals aged ≥ 18 years presenting with a thyroid nodule measuring 3 to 15 mm in maximal diameter, with cytology samples classified as Bethesda V (suspicious for malignancy) or VI (malignant), treated at the thyroid services of one public hospital (*Irmandade da Santa Casa de Misericórdia de São Paulo*) and two private hospitals (*Hospital Samaritano-Higienópolis de São Paulo* and *A Beneficência Portuguesa de São Paulo*). Patients were excluded if their anatomopathological examinations were not available, if they had serum calcitonin values of ≥ 10 pg/mL, or if they had a current diagnosis of non-thyroid neoplasia.

### Variables

The clinical variables evaluated were age, sex, and history of neoplasms or thyroid diseases. The ultrasonographic characteristics of the nodules assessed included size, echogenicity, composition, shape, margins, echogenic *foci*, vascularization, extrathyroidal invasion, location, lymph node metastasis, and Thyroid Imaging, Reporting and Data System of the American College of Radiology (TI-RADS ACR classification) (^34^). The histological characteristics evaluated were size, histological type and subtype, extrathyroidal invasion, vascular invasion, lymph node metastasis, TNM staging, and ATA RRP (^12^). Patients classified as low RRP were considered to have tumors with indolent behavior, whereas those classified as intermediate/high-risk were considered to have tumors with aggressive behavior.

### Cervical ultrasound

To minimize inter-observer variability, all ultrasound examinations were conducted by a single examiner at each of the three institutions. Any uncertain findings were discussed among the three sonographers until a consensus was reached.

### miRs selection

The panel consisted of miR-146b-5p, miR-204, and miR-16. For miR selection, we aimed to include one miR with high expression associated with poor prognosis and another whose low expression is linked to unfavorable outcomes. miR-146b-5p is the most extensively studied miR in relation to aggressive PTC behavior (^22,24,26,27^). Moreover, data from a previous study conducted by our group demonstrated a correlation between serum miR-146b-5p levels and PTC prognosis (^35^). In contrast, miR-204 was selected for its protective role, with strong evidence indicating that its downregulation is related to tumor aggressiveness in PTC (^28,29^). Based on prior studies involving liquid biopsy, miR-16 was used as a reference gene (^21,30,36^).

### Molecular analysis

Peripheral venous blood (4 mL) was collected in ethylenediamine tetraacetic acid (EDTA) tubes for serum miRNA analysis from both the AS and surgery groups at the beginning of the study, prior to any surgical intervention. The samples were centrifuged within 60 minutes of collection at 1,900 rpm for 15 minutes at 4 °C to obtain serum, which was then stored at -80 °C. RNA extraction was performed using the miRNeasy Micro Kit (Qiagen, Germany), as outlined by the manufacturer. The quantity and integrity of the extracted RNA were evaluated using a NanoDrop 2000 (Thermo Scientific, USA). Samples with a 260:280 ratio of 1.4 were considered to have satisfactory purity. Approximately 400 ng of RNA was converted to 30 µL of cDNA using the miScript II RT Kit (Qiagen, Germany).

Real-time polymerase chain reaction (7500, Applied Biosystems, USA) was performed using commercially available Qiagen custom primers for miR-146b-5p, miR-204, and miR-16, as well as the miScript SYBR Green PCR Kit (Qiagen, USA). For each assay, 1.5 µL of cDNA was used. The real-time polymerase chain reaction conditions consisted of 40 cycles: 15 minutes at 95 °C for activation, 15 seconds at 94 °C for denaturation, 30 seconds at 55 °C for annealing, and 34 seconds at 70 °C for extension, followed by a melting curve. Each reaction was conducted in duplicate. Cycle threshold values above 40 were considered non-significant and excluded.

### Cost estimates

The costs for molecular analysis were limited to direct variables (costs of reagents and controls) and indirect laboratory running costs (disinfection, error corrections, and repetitions). Investment costs, such as laboratory space, equipment, training, quality assurance, accreditation, and labor expenses were not included.

### Statistical analysis

We used IBM Statistical Package for the Social Sciences (SPSS) software (version 20.0) and MedCalc Online (version 22.012). Chi-squared and Fisher’s exact tests were used for categorical variables, while Mann-Whitney and Kruskal-Wallis tests were applied to continuous variables. Relative quantification of the molecular data was performed using the delta cycle threshold method. The cut-off value for miR expression was determined using the ROC curve, considering the best value of the Youden index capable of differentiating RRP into low or intermediate/high risk. Statistical significance was set at p < 0.05. We used G*Power software (version 3.1.9.7) to calculate the statistical power for the analyzed study sample. The cut-off values for miRs established in the Surgery Group were used to classify the AS Group. Based on these cut-off points, two test models were developed: a high-sensitivity test and a high-specificity test.

### Ethics and informed consent

Written informed consent was obtained from each participant prior to enrollment. Additionally, the study was approved by the research ethics committees of *Irmandade da Santa Casa de Misericórdia de São Paulo* (CAAE no. 30566920.4.0000.5479), *A Beneficência Portuguesa de São Paulo* (CAAE no. 30566920.4.3001.5483), and *Hospital Samaritano - Higienópolis de São Paulo* (CAAE no. 73456317.4.0000.5487), in addition to adhering to the principles set out in the Declaration of Helsinki.

## RESULTS

### Sample description

Of the 51 patients, 21 (41.2%) underwent surgery at diagnosis, while 30 (58.8%) comprised the AS Group. During follow-up, five patients (16.6%) transitioned from the AS Group to the Surgery Group. Molecular analysis could not be performed in seven patients in the AS Group due to logistical reasons; however, these patients remained in the study, except for one patient who died of unrelated causes.

The characteristics of the patients and nodules are described in **Table 1**. Overall, 76% of the patients were women, and 55% were aged 40 to 59 years. Patients in the Surgery Group were younger than those in the AS Group, with a mean age of 41.9 ± 7.9 and 53.5 ± 12.6 years, respectively (p < 0.001). Additionally, the Surgery Group had nodules exhibiting more malignant characteristics, such as a taller-than-wide shape (p = 0.016), lobulated or irregular margins (p = 0.047), and punctate echogenic foci (p = 0.027).

**Table 1:** Characteristics of the study sample (n = 51).

Variables	Total	AS Group	Surgery Group	p-value	Test
n	51	30	21		-
Hospital				0.739	Fisher’s exact test
Public	12 (24)	8 (27)	4 (19)		
Private	39 (76)	22 (73)	17 (81)		
Sex				0.196	Fisher’s exact test
Male	12 (24)	5 (17)	7 (33)		
Female	39 (76)	25 (83)	14 (67)		
Age, years				0.000	Fisher’s exact test
≤ 39	12 (24)	3 (10)	9 (43)		
40-59	28 (55)	16 (53)	12 (57)		
≥ 60	11 (21)	11 (37)	0		
Time (months) of follow-up under AS				-	-
≤ 15	7 (14)	7 (23)	-		
16-25	4 (8)	4 (13)	-		
26-50	10 (20)	10 (33)	-		
≥ 51	9 (18)	9 (30)	-		
ACR TI-RADS				0.445	Fisher’s exact test
4	8 (16)	6 (20)	2 (10)		
5	43 (84)	24 (80)	19 (90)		
Age, years	48.7 ± 12.3	53.5 ± 12.6	41.9 ± 7.9	0.000	T-test
Time (months) of follow-up under AS	36.4 ± 25.8	36.4 ± 25.8	-	-	-

Results expressed as n, n (%) or mean ± standard deviation.

AS: active surveillance; ACR TI-RADS: American College of Radiology Thyroid Imaging Reporting and Data System.

### Surgery Group

The histological characteristics of the patients who underwent surgery are detailed in **Table 2**. In this group, 22 (88%) patients had nodules measuring < 10 mm in diameter. Lymph node metastasis was identified in seven patients, multifocality in six, and extrathyroidal invasion in five, including one case of macroscopic extrathyroidal invasion. There was one instance of the aggressive subtype (high cells), and another case was classified as having high RRP.

**Table 2:** Anatomopathological characteristics of the patients who underwent surgery (n = 25)

Variables	
Reasons for indicating surgery at diagnosis	
Patient’s preference	5 (24)
Contact with the anterior thyroid capsule	6 (29)
Contact with the posterior thyroid capsule	2 (9)
Contact with the trachea	2 (9)
Lymph node metastasis	1 (5)
Location in the isthmus	1 (5)
Suspicion of extrathyroidal invasion	1 (5)
Psychiatric disorder	1 (5)
Graves’s disease	2 (9)
Agressive subtype on cytology	0
Reasons for indicating surgery after active surveillance	
Patient’s preference	2 (40)
Contact with the anterior thyroid capsule	2 (40)
Contact with the posterior thyroid capsule	1 (20)
Type of surgery	
Partial thyroidectomy	8 (32)
Total thyroidectomy	15 (60)
Total thyroidectomy with central neck dissection	1 (4)
Total thyroidectomy with lateral neck dissection	1 (4)
Nodule size (mm)	
< 6	7 (28)
6-10	15 (60)
≥ 11	3 (12)
Papillary thyroid carcinoma histological type	
Histological subtype	
Classic	22 (88)
Follicular	2 (8)
Tall cells	1 (4)
Multifocality	6 (24)
Extrathyroidal invasion	5 (20)
Lymph node metastasis	7 (28)
Distant metastasis	0
TNM	
T1AN0	18 (72)
T1AN1A	3 (12)
T1BN0	1 (4)
T1BN1A	1 (4)
T1BN1B	1 (4)
T3BN0	1 (4)
Risk of recurrence and/or persistence^*^	
Low	16 (64)
Intermediate	8 (32)
High	1 (4)

* According to the American Thyroid Association risk stratification system (12).

Results expressed as n (%).

Regarding indications for surgery at the time of diagnosis, six patients were referred for surgery because ultrasound assessment showed contact with the anterior thyroid capsule, and two were referred due to contact with the posterior capsule. Anatomopathological results did not confirm extrathyroidal invasion in any of these cases. One patient was referred for surgery based on suspected extrathyroidal invasion of the perithyroidal muscles on ultrasound and indeed presented with macroscopic extrathyroidal invasion. Among the five patients who transitioned from AS to surgery, two cases were due to suspected invasion of the anterior thyroid capsule, which was not confirmed later, and one was due to suspected invasion of the posterior capsule, which was confirmed postoperatively.

Regarding patient preference, five patients opted for surgery at diagnosis, while another five chose to switch to surgery at a later stage. Compared to those who remained in AS, the patients who either preferred or switched to surgery were younger, with mean ages of 54.1 ± 12.8 and 40.3 ± 6 years, respectively (p < 0.001).

### Active Surveillance Group

The mean follow-up duration in the AS Group was 36.4 ± 25.8 months, whereas the mean time before migration to surgery was 22.2 ± 12.2 months. The size of the nodules remained stable over time, with no patient demonstrating significant nodule growth. Nodule volume showed greater fluctuation compared to diameter, albeit tended to decrease by the end of the follow-up period. No cases of lymph node or distant metastases were observed during AS, and one patient exhibited an increase in the number of nodules from one to four.

### Molecular analysis

Molecular analysis was conducted in 16 patients classified as low RRP, eight as intermediate risk, one as high risk, and in 23 patients from the AS Group. miR-16, used as the reference gene, demonstrated consistent expression levels among patients, exhibiting a relatively low standard deviation (mean absolute cycle threshold value of 32.35 ± 4.3).

**Figure 2:**
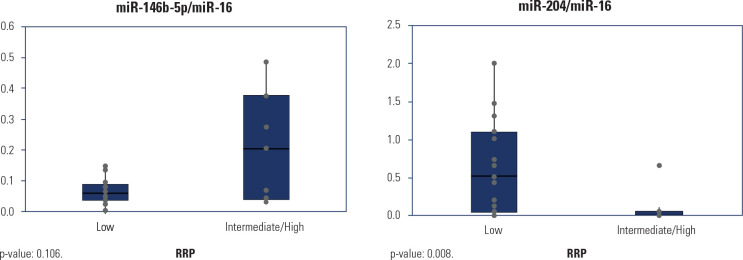
Levels of miR-146b-5p/miR-16 and miR-204/miR-16 in patients with low and intermediate/high risk of recurrence and/or persistence according to the American Thyroid Association risk stratification system.

A statistically significant difference in miR-204 levels was observed between the low and intermediate/high RRP groups (p = 0.008; **Table 3** and **Figure 2**). Using the optimal Youden index, the cut-off values for miR-146b-5p/miR-16 and miR-204/miR-16 were determined to be 0.1757 and 0.02223, respectively, both of which significantly differentiated RRP groups. The miR-204/miR-16 analysis demonstrated strong statistical power (0.972), as the required sample size to detect the area under the curve (AUC) > 0.8 was 18 cases, and our study included 23 patients (AUC = 0.833). Conversely, the analysis of miR-146b-5p/miR-16 yielded an AUC of 0.24 with 22 valid cases, resulting in a test power of 0.594. The expression levels of these miRNAs were not associated with any specific ultrasonographic or histological features.

**Table 3:** Levels of miR-146b-5p/miR-16 and miR-204/miR-16 according to ultrasound and anatomopathological characteristics of patients who underwent surgery

Nodule characteristics	Median (minimum-maximum)	
	miR-146/miR-16 (n = 42)	miR-204/miR-16 (n = 42)
Multifocality		
No	0.088 (0.001-0.485)	0.156 (0-2.274)
Yes	0.043 (0.001-24.761)	0.014 (0-0.653)
p-value	0.426	0.080
Lymph node metastasis		
No	0.081 (0.001-24.761)	0.126 (0-2.274)
Yes	0.036 (0.036-0.036)	0.053 (0.053-0.053)
p-value	0.429	1.000
Composition		
Solid/almost completely solid	0.08 (0.001-24.761)	0.089 (0-2.274)
p-value	-	-
Echogenicity		
Isoechoic	0.025 (0.001-0.081)	0.337 (0.027-0.441)
Hypoechoic	0.088 (0.001-24.761)	0.053 (0-2.274)
p-value	0.111	0.747
Shape		
Wider-than-tall	0.09 (0001-24.761)	0.085 (0-2.274)
Taller-than-wide	0047 (0.001-0.155)	0.125 (0-2.007)
p-value	0.261	0.440
Margin		
Smooth/ill-defined	0.084 (0.005-0.198)	0.323 (0.001-2.274)
Lobulated/ irregular	0.074 (0.001-24.761)	0.044 (0-2.007)
p-value	0.622	0.362
Echogenic foci		
No	0.088 (0.001-24.761)	0.156 (0.001-2.274)
Punctate echogenic foci	0.068 (0.005-0.485)	0.044 (0-2.007)
p-value	0.678	0.651
Vascularity		
Peripheral	0.067 (0.011-0.485)	0.125 (0-2.274)
Central	0.068 (0.001-0.093)	0.156 (0.001-1.102)
p-value	0.527	0.921
Extrathyroidal invasion		
No	0.08 (0.001-24.761)	0.048 (0-2.274)
Yes	0.095 (0.025-0.485)	0.547 (0.018-1.474)
p-value	0.757	0.287
ACR TI-RADS		
4	0.051 (0.001-0.107)	0.021 (0.001-0.653)
5	0.086 (0.001-24.761)	0.126 (0-2.274)
p-value	0.087	0.217
Anatomopathological characteristics	Median (minimum-maximum)	
	miR-146/miR-16 (n = 42)	miR-204/miR-16 (n = 42)
Nodule size (mm)		
< 6	0.09 (0.005-0.376)	0.126 (0.005-2.007)
6-10	0.062 (0.025-0.275)	0.319 (0-1.474)
≥ 11	0.068 (0.001-0.485)	0.02 (0.014-0.027)
p-value	0.861	0.649
Histological subtype		
Classic	0.079 (0.005-0.485)	0.284 (0-2.007)
Follicular	0.034 (0.001-0.067)	0.112 (0.027-0.198)
Tall cells	0.068 (0.068-0.068)	0.014 (0.014-0.014)
p-value	0.454	0.726
Multifocality		
No	0.083 (0.001-0.376)	0.162 (0.001-2.007)
Yes	0.053 (0.025-0.485)	0.044 (0-0.653)
p-value	0.449	0.403
Extrathyroidal invasion		
No	0.067 (0.001-0.275)	0.198 (0-2.007)
Yes	0.376 (0.043-0.485)	0.011 (0.005-0.653)
p-value	0.191	0.188
Lymph node metastasis		
No	0.062 (0.001-0.485)	0.162 (0-2.007)
Yes	0.096 (0.036-0.275)	0.053 (0.001-1.474)
p-value	0.332	0.871
TNM		
T1AN0	0.057 (0.001-0.376)	0.198 (0-2.007)
T1AN1A	0.148 (0.096-0.204)	0.732 (0.001-1.474)
T1BN0	0.485 (0.485-0.485)	-
T1BN1A	0.068 (0.068-0.068)	0.014 (0.014-0.014)
T1BN1B	0.036 (0.036-0.036)	0.053 (0.053-0.053)
T3BN0	-	0.018 (0.018-0.018)
p-value	0.117	0.879
Risk of recurrence and/or persistence^*^		
Low	0.062 (0.001-0.148)	0.511 (0-2.007)
Intermediate/high	0.204 (0.036-0.485)	0.01 (0.001-0.653)
p-value	0.106	0.008

* According to the American Thyroid Association risk stratification system (12).

ACR TI-RADS: American College of Radiology Thyroid Imaging Reporting and Data System.

RRP: risk of recurrence/persistence.

Source: Haugen and cols. (12).

**Figure 3:**
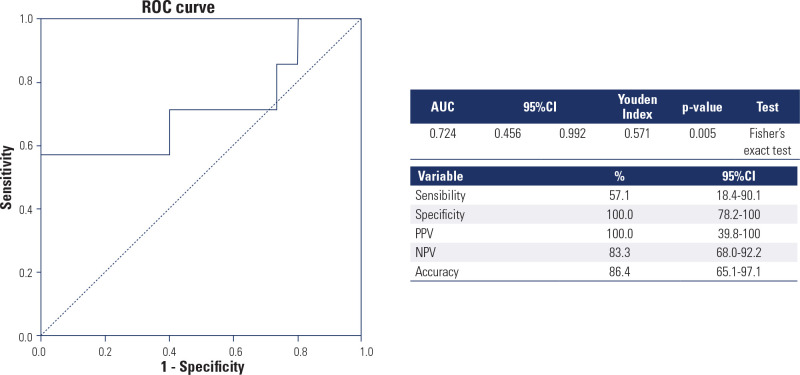
Receiver Operating Characteristic curve generated from the miR-146b-5p/miR-16 cut-off point of 0.1757 to differentiate patients with low risk from those with intermediate/high risk of recurrence and/or disease persistence according to the American Thyroid Association risk stratification system.

Source: Haugen and cols. (12).

ROC: Receiver Operating Characteristic; AUC: area under curve; 95%CI: 95% of confidence interval; PPV: positive predictive value; NPV: negative predictive value.

**Figure 4:**
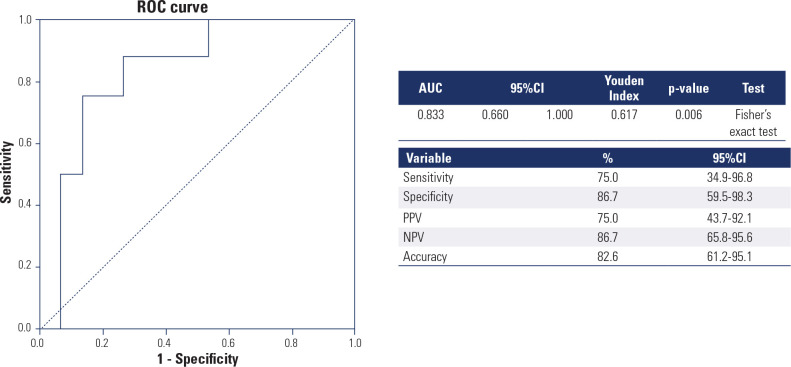
Receiver Operating Characteristic curve generated from the miR-204/miR-16 cut-off point of 0.02223 to differentiate patients with low risk from those with intermediate/high risk of recurrence and/or disease persistence according to the American Thyroid Association risk stratification system.

Source: Haugen and cols. (12).

ROC: Receiver Operating Characteristic; AUC: area under curve; 95%CI: 95% of confidence interval; PPV: positive predictive value; NPV: negative predictive value.

Upregulation of miR-146b-5p/miR-16 resulted in a sensitivity of 57.1%, specificity of 100%, positive predictive value (PPV) of 100%, negative predictive value (NPV) of 83.3%, and accuracy of 86.4% for distinguishing intermediate/high RRP from low-risk RRP patients (Youden index: 0.571; p = 0.005) (**Figure 3**). Downregulation of miR-204/miR-16 presented a sensitivity of 75%, specificity of 86.7%, PPV of 75%, NPV of 86.7%, and accuracy of 82.6% for differentiating intermediate/high RRP from low-risk RRP patients (Youden index: 0.617; p = 0.006) (**Figure 4**).

### Test models

**Figure 5:**
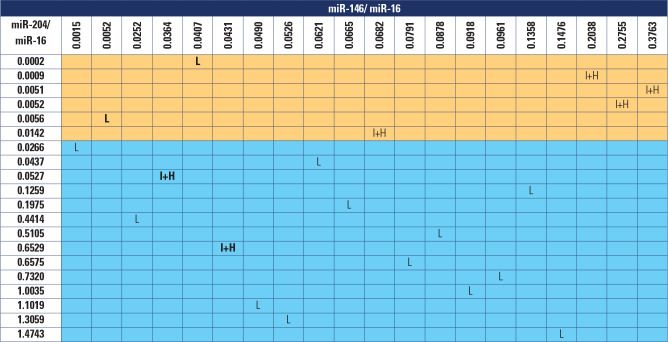
Model 1 molecular test applied to patients undergoing surgery.

Source: Haugen and cols. (12).

**Figure 6:**
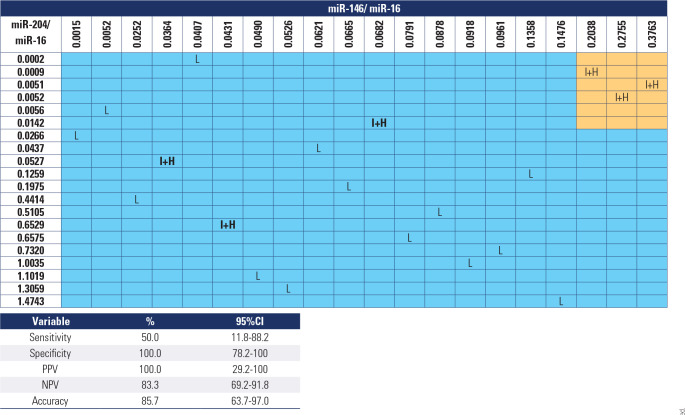
Model 2 molecular test applied to patients undergoing surgery.

Source: Haugen and cols. (12).

Based on these cut-off points, two test models were developed. In the first model, the objective was to obtain a high-sensitivity test so that only cases with indolent behavior would be included in the AS Group. In this model, the miR-204/miR-16 analysis was sufficient, providing good sensitivity (75%) and high NPV (86.7%) (**Figure 5**). The second model aimed to develop a highly specific test to ensure that only patients with aggressive behavior would be referred for surgery. The optimal combination in this case was miR-146b-5p/miR-16 together with downregulation of miR-204/miR-16, yielding a sensitivity of 50%, specificity of 100%, PPV of 83.3%, NPV of 100%, and accuracy of 85.7% (**Figure 6**).

Orange: patients who tested positive; blue: patients who tested negative. Cases that would be incorrectly classified according to the risk of recurrence and/or persistence are highlighted in bold. L: low risk of recurrence and/or persistence; I+H: intermediate or high risk of recurrence and/or persistence according to the American Thyroid Association risk stratification system.

Orange: patients who tested positive; blue: patients who tested negative. Cases that would be incorrectly classified according to the risk of recurrence and/or persistence are highlighted in bold. L: low risk of recurrence and/or persistence; I+H: intermediate or high risk of recurrence and/or persistence according to the American Thyroid Association risk stratification system.

95%CI: 95% of confidence interval; PPV: positive predictive value; NPV: negative predictive value.

By applying the first model to patients in the AS Group, we found nine patients with downregulated miR-204/miR-16. When applying the second model, we identified two suspected cases of aggressive behavior (**Table 4**). Analysis of these two cases showed that one patient had been in AS for 5.8 years, progressing with an increase in the number of nodules from one to four. In the other case, the patient had been under follow-up for 2.5 years with a stable nodule.

**Table 4:** Application of model 2 molecular test in patients from the Active Surveillance Group who migrated to surgery and patients from the Active Surveillance Group who remained in active surveillance until the end of the study

miR204/ miR16	miR146/ miR16	Test result	Patients in the AS Group who migrated to surgery		Patients in the AS Group who remained in AS
			Low RRP	Intermediate/ high RRP	-
< 0.02223	< 0.1757	Negative	0	0	7
< 0.02223	≥ 0.1757	Positive	0	1	2
≥ 0.02223	< 0.1757	Negative	2	0	9
≥ 0.02223	≥ 0.1757	Negative	0	0	1

* RRP according to American Thyroid Association risk stratification system (12).

AS: active surveillance; RRP: risk of recurrence/persistence.

Of the five patients who were transferred from AS to surgery, three underwent molecular analysis. Among these, two were classified as low risk and one as intermediate risk (**Table 4**). Using the first model, two low-risk cases were identified. By adding the analysis of the second model, only the case classified as intermediate risk was identified. Therefore, surgery could have been avoided in two patients with indolent behavior.

## DISCUSSION

Differentiating between indolent and aggressive tumors remains the pivotal challenge in implementing AS for thyroid cancer. Existing molecular tests, primarily designed to distinguish between benign and malignant thyroid nodules and guide targeted therapies have offered some insights (^37^). Recently, molecular profiles indicating high risk, such as the coexistence of the BRAFV600E mutation with mutations in TERT promoter regions, have been significantly associated with metastasis in patients with differentiated thyroid cancer, thereby enhancing their prognostic value for informing surgical approaches and the indications for radioiodine therapy (^13,38^). However, there is a noticeable gap in the literature regarding molecular tests specifically aimed at evaluating thyroid tumor behavior to identify patients who might avoid surgery due to indolent disease. Our study demonstrated that serum expression levels of miR-146b-5p/miR-16 and miR-204/miR-16 could distinguish patients with low RRP from those at intermediate/high risk. Furthermore, we developed test models based on these miRs that proved effective in selecting patients for AS, thereby avoiding unnecessary surgery.

The increased expression of miR-146b in PTC compared to normal tissues has been well documented and is identified as an indicator of aggressiveness (^36,39^). This expression is notably higher in classic PTC subtypes than in follicular variants and is similar between classic and tall cell subtypes (^29^). In fact, a well-documented correlation exists between miR-146b expression with lymph node metastasis and extrathyroidal invasion (^39,40^). Although our study did not find a significant difference in miR-146b-5p/miR-16 expression between subtypes or a direct association with histological findings, a significant association was observed with the RRP classification, encapsulating all these variables (e.g., aggressive subtype, lymph node metastasis, and extrathyroidal invasion). The lack of statistical significance for individual histological characteristics may be attributed to the small sample size. Zhang and cols. (^36^) reported an upregulation of these miRs in PTC patients compared to those with multinodular goiter and healthy individuals, a downregulation post-thyroidectomy, and a correlation with lymph node metastasis and extrathyroidal invasion; however, the authors did not evaluate RRP.

Evidence has suggested that miR-204 is one of the most significantly downregulated miRs in PTC and associated with aggressive histological features (^41,42^), such as extrathyroidal invasion, lymph node metastasis, high cell subtype, TNM staging, and the BRAFV600E mutation (^29^). Similar to miR-146b, we noted a significant association between the downregulation of miR-204/miR-16 and the recurrence and progression risk, albeit without correlation to specific histological features.

We developed two test models: a rule-out test designed to predict tumors with indolent behavior, requiring high NPV and sensitivity, and a rule-in test aimed at identifying malignancy, necessitating high PPV and specificity (^43,44^). Analyzing miR-204/miR-16 expression proved sufficient as a rule-out test, while the combined analysis of miR-146b-5p/miR-16 and miR-204/miR-16 served effectively as a good rule-in test.

Regarding indications for AS, age emerged as the sole differing factor between the AS and surgery groups. Indications for surgery were significantly more prevalent among younger patients, likely corroborating the findings of Ito and cols. (^45^), who reported that an age of 40 years or younger is associated with tumor progression in PTC. Brito and cols. (^46^) categorized patients aged 18 years as inappropriate for AS, 18 to 60 years as appropriate, and > 60 years as ideal candidates. The decision to undergo surgery also corroborates the findings of Sawka and cols. (^16^), who found that younger individuals tend to prefer surgical management.

Consistent with existing large AS studies that reported low rates of disease progression in low-risk PTC tumors (^5-10,14,47^), our study observed no instances of nodule growth above 3 mm or the emergence of lymph node or distant metastasis during follow-up.

Despite the promising results, this study’s limitations include a relatively small sample size, potentially affecting the findings’ generalizability. Future research could benefit from miRNA selection via next-generation sequencing to increase robustness. Another challenge lies in the difficulty of comparing cut-off values used in this study with those from other studies due to methodological differences in processing and analysis. Additionally, the developed testing models, along with their cost-effectiveness, require validation in a larger cohort to verify their clinical applicability.

We propose that analyzing miR-204/miR-16 expression can serve as an exclusion test to identify patients with indolent PTC for AS. Conversely, the combined analysis of miR-146b-5p/miR-16 and miR-204/miR-16 can function as an inclusion test to refer patients exhibiting aggressive PTC behavior for surgery.

Implementing these tests could significantly impact the management of low-risk tumors and reduce the economic burden of thyroid nodules in Brazil (^48^). We estimate that molecular analysis of miR-146b-5p and miR-204 would incur a cost of US$ 90.00 per patient, factoring in material costs but excluding labor expenses. According to a previous study by our group, the cost of AS for low-risk PTC is lower than that of surgery at diagnosis, extending through 10, 20, and 30 years of follow-up (^49^). The overall cost of AS treatment should continue to be lower than that of surgical treatment after including the cost of molecular analysis.

In conclusion, we developed a cost-effective serum test that may assist in clinical decision-making regarding AS eligibility. Nevertheless, it requires further validation through larger independent cohorts to confirm its clinical utility.
